# Chinese Pangolins in China Demonstrate Regional Differences in Burrow Habitat Selection

**DOI:** 10.3390/ani15142093

**Published:** 2025-07-16

**Authors:** Dongling Liang, Xinrui Tang, Yilong Chen, Fei Xi, Shibao Wu, Fuhua Zhang

**Affiliations:** Guangzhou Key Laboratory of Subtropical Biodiversity and Biomonitoring, School of Life Sciences, South China Normal University, Guangzhou 510631, China; 15817155861@163.com (D.L.); txr2430105963@163.com (X.T.); yilong1103@163.com (Y.C.); 17836937824@163.com (F.X.)

**Keywords:** China, habitat selection, human disturbance, *Manis pentadactyla*

## Abstract

Knowledge of the habitat characteristics of endangered species is an important basis for in situ conservation, release-site selection, and habitat modification. Although the Chinese pangolin (*Manis pentadactyla*) is one of the world’s most endangered species, little is known about its habitat preferences, and the results of past studies differ greatly. To clarify the habitat characteristics of the Chinese pangolin, we conducted habitat surveys in Guangdong, Jiangxi, and Zhejiang provinces of China using the transect method. A total of 520 burrow sites of Chinese pangolins were recorded in three study areas. The resulting data were analyzed using a generalized additive model, principal coordinate analysis, and Kruskal–Wallis tests. Nine ecological factors (elevation, slope, soil type, canopy coverage, surface coverage, number of trees, number of logs, tree diameter at breast height, and distance to a settlement) were found to affect pangolins’ distribution. Burrows were preferentially distributed at elevations of 50–150 m (62.3%), in silty soil (88.1%), on 20–40° slopes (83.3%), within young and medium-aged broadleaved forests with a canopy coverage exceeding 70% (65.8%), and close to water (less than 300 m). Among the three study regions, pangolin habitats differed significantly in seven environmental factors: elevation, canopy coverage, surface coverage, number of trees, distance to water, distance to a road, and distance to a settlement. Our findings imply that the Chinese pangolin appears to tolerate a broad range of ecological characteristics; however, food resources may be the key factor affecting its habitat selection, and other factors may indirectly affect its distribution by affecting food abundance. Finally, aside from hunting, a low level of human disturbance does not affect the presence of this species.

## Simple Summary

The Chinese pangolin (*Manis pentadactyla*) is one of the world’s most endangered species. A thorough understanding of its habitat characteristics is critical for effective conservation, yet existing research has not reached a consensus on these features. To address this gap, we conducted habitat surveys across three Chinese provinces—Guangdong, Jiangxi, and Zhejiang—to investigate the habitat preferences of the Chinese pangolin and assess potential regional variations. Our findings revealed that pangolin burrows were predominantly distributed at elevations of 50–150 m (62.3%), in silty soil (88.1%), on 20–40° slopes (83.3%), within young and medium-aged broadleaved forests with a canopy coverage exceeding 70% (65.8%), and close to water (less than 300 m). Notably, habitat characteristics varied significantly across the study regions. Additionally, we found that pangolins can tolerate moderate human disturbance, excluding direct hunting pressure. This study provides valuable reference data to support in situ conservation efforts, inform release site selection, and guide habitat restoration initiatives for the Chinese pangolin.

## 1. Introduction

Habitat selection refers to an animal’s preferential use of specific habitats that provide essential resources for survival and reproduction [1]. Habitat selection is an important focus of ecological and evolutionary studies [2], as understanding species’ habitat characteristics provides an important reference for conservation and management efforts such as planning and designing protected areas [3], selecting species’ release sites, and modifying and restoring habitat [4,5]. Many studies on animal habitat selection focus on a specific study area. But for widely distributed species, studies in localized areas do not allow clear identification of the universally important resources [6].

The Chinese pangolin (*Manis pentadactyla*; order Pholidota) is widely distributed south of the Yangtze River in China [7]; in Southeast Asian countries such as Vietnam [8], Laos [9], Myanmar [10], and Thailand [11]; and in Bangladesh [12], India [13], Bhutan [14], and Nepal [15]. However, their populations have declined sharply due to overharvesting and habitat loss, and this species is classified as critically endangered on the IUCN Red List [16]. If current trends continue without any conservation measures, this species will soon face extinction. Protecting this species is therefore of urgent importance.

Understanding Chinese pangolins’ habitat characteristics is a prerequisite for in situ and ex situ conservation. However, their special morphological, biological, and ecological characteristics make it extremely difficult to investigate and monitor them in the field [17]. Only a few studies have been conducted on the habitats of this species, mainly in Nepal [15,18,19,20,21,22,23], and a few in China [24,25,26,27] and Bhutan [14]. Although such studies have generally assumed that environmental factors—including elevation, slope, aspect, canopy coverage, forest type, and distance to water, settlements, and roads—are important ecological factors that may affect habitat selection, conclusions on the most important factors and the preferences of pangolins have been inconsistent among studies. For example, reported elevation preferences for this species vary from 100–200 m [26] to over 2000 m above sea level (a.s.l.) [28], with some studies suggesting below 1000 m [20,22,23,24,25] and others 1000–2000 m [14,19,21,29,30] or higher [28]. Thus, the key ecological factors affecting the distribution of these pangolins and their preferences for various habitat characteristics remain unclear. This lack of clarity may be because this species has a wide distribution range [31], whereas studies have been limited to specific areas; thus, their conclusions have not been broadly representative or generalizable. This implies a need for habitat studies at larger scales; additional sites, national, and international data will enable conservationists, protected area managers, practitioners, and researchers to better understand the habitat requirements of this species, and facilitate the identification and prioritization of key sites for the conservation of the Chinese pangolin.

In this study, the distribution and habitat characteristics of Chinese pangolins were studied in Guangdong, Jiangxi, and Zhejiang provinces of China, in three important distribution areas of the Chinese pangolin, at different latitudes. This study aims to (1) investigate the habitat characteristics of Chinese pangolins, (2) identify key ecological factors influencing their habitat selection, and (3) assess potential regional differences in habitat characteristics. Our results not only provide essential baseline data on the distribution and habitat characteristics of this species but also offer crucial data that can inform future conservation efforts such as habitat protection, habitat restoration, reserve planning, and release-site selection.

## 2. Materials and Methods

### 2.1. Study Area

**Figure 1:**
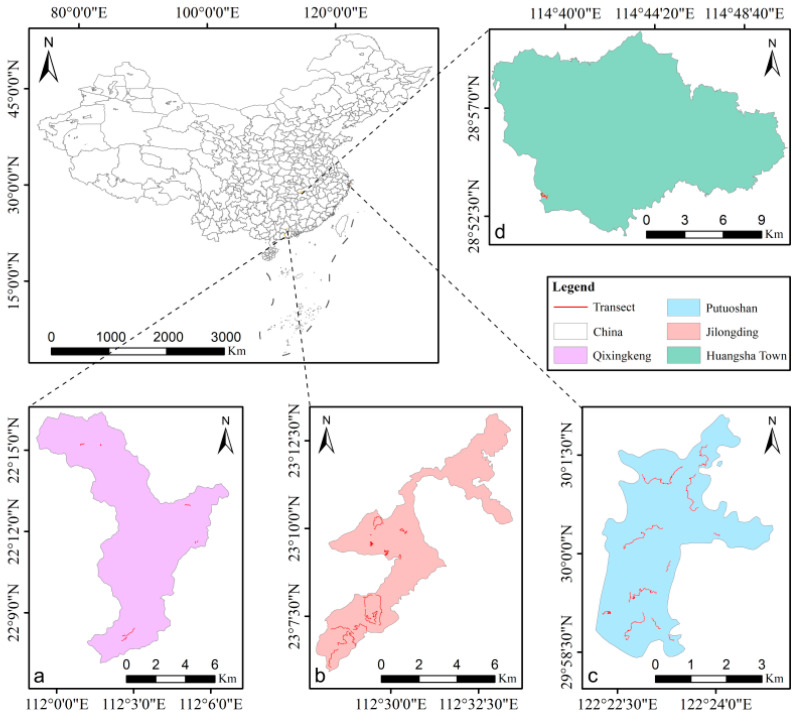
Map of the study areas showing the transects. (**a**,**b**) are located in Guangdong Province, (**c**) is located in Zhejiang Province, and (**d**) is located in Jiangxi Province.

This study was conducted in Guangdong Province, Jiangxi Province, and Zhejiang Province in China (Figure 1). The study areas included the following: in Guangdong, Enping Qixingkeng Provincial Nature Reserve (hereafter, Qixingkeng), situated in Jiangmen City (111°59′54″–112°07′30″ E, 22°06′56″–22°16′14″ N, 50–1010 m a.s.l., 78.9 km^2^), Zhaoqing Jilongding Municipal Nature Reserve and its surrounding areas (hereafter, Jilongding), located in Zhaoqing City (112°23′56″–112°33’41″ E, 23°5′3″–23°11′30″ N, 10–1000 m a.s.l., approximately 30 km^2^), and Xiagaoli Village (hereafter, Xiagaoli, ~5 km^2^) of Huangsha Town (114°37′–114°49′ E, 28°52′–28°59′ N, 260–1200 m a.s.l.); in Jiangxi, Xiushui County; and in Zhejiang, on Putuoshan Island (hereafter, Putuoshan), located in Zhoushan City (122°21′6″–122°24′9″ E, 29°58′3″–30°2′3″ N, 0–286 m a.s.l., 12 km^2^). In Guangdong, the study area is designated as a protected zone, yet it still experiences moderate anthropogenic disturbances, including sporadic poaching incidents. In Jiangxi, the research site comprises privately owned forestland subjected to intermediate levels of human disturbance, with occasional logging activities observed. In contrast, the study area in Zhejiang is a tourist island where, despite some human pressures, understory economic exploitation and poaching have been completely eradicated.

### 2.2. Pangolin Distribution and Habitat Factors

As the Chinese pangolin is exclusively a burrowing animal [32], the spatial distribution of its burrows serves as an effective indicator for studying the population and habitat characteristics of this species [17,24]. Between October 2021 and December 2023, the transect method was used to conduct field surveys in the three study regions. Transect locations were strategically determined based on three primary criteria: (1) spatial distribution within the study area, (2) vegetation heterogeneity, and (3) accessibility. This design approach was implemented to minimize spatial autocorrelation between sampling transects. In total, 53 transects were established (Supplement Table S1), each with a width of 6–10 m (adjusted based on local vegetation density), an average length of 1.23 ± 0.76 km, and a total length of 65.01 km (Figure 1). During the surveys, we searched for pangolin burrows along these transects, and control sites were set every 50–100 m in areas without burrows. Following Kang [33], we established microhabitat quadrats and recorded the following environmental factors: longitude and latitude, elevation, aspect, slope, forest type, soil type, canopy coverage, surface coverage, number of logs, number of stumps, number of trees, tree diameter at breast height (DBH), distance to trees, distance to water, distance to a road, and distance to a settlement. The methods used to measure each factor are given in Supplement Table S2.

### 2.3. Data Analysis

Generalized additive models (GAMs) are widely used to study relationships between organisms and their environment because of the models’ ability to capture linear and nonlinear changes in important variables [34,35]. In this study, the response variable was the distribution of pangolins and the explanatory variables were the observed environmental factors. GAMs were conducted following a previous study [34], and the distribution of, and environmental factors associated with, Chinese pangolins were analyzed via regression to identify the key environmental factors affecting habitat selection and their effects on the distribution of this species.

Prior to modeling, Pearson correlation analysis was performed on all habitat factors to avoid overfitting the model. For environmental factors, only those thought to have the greatest impact on pangolin distribution (correlation values |r| > 0.7) were retained during modeling [27]. A GAM was used to identify habitat variables that significantly affected the distribution of this species. To this end, forward selection was used; that is, starting from an empty model, we added each candidate factor to the GAM separately and evaluated the goodness of fit of the model using the Akaike information criterion (AIC). The factor that led to the best model was retained, and then the above process was repeated continuously until the model could not be significantly improved by the addition of any other factor [34]. The environmental factors included in the model with the lowest AIC value were considered the key factors affecting the distribution of Chinese pangolins. Finally, the model was employed to elucidate how each of the key factors influenced the observed distribution of pangolin burrows.

Permutational multivariate analysis of variance (PERMANOVA) was used to evaluate overall differences between the pangolin habitats in the three study areas. Principal coordinates analysis (PCoA), based on Bray–Curtis distance, was used to demonstrate the degree of separation of habitats between the different regions. Since the Kolmogorov–Smirnov and Shapiro–Wilk tests indicated non-normal distribution of data, the Kruskal–Wallis test was performed to test the overall differences among the three study regions (Supplement Table S3). For factors with significant differences (*p* < 0.05), post hoc pairwise comparisons were conducted to analyze the differences between single factors in the three different regions.

GAM, PERMANOVA, and PCoA were performed using the “mgcv ver. 1.9-3”, “vegan ver. 2.7-1”, “tidyverse ver. 2.0.0”, and “ape ver. 5.7-1” packages in R ver. 4.3.3, respectively. The Kruskal–Wallis test was performed in SPSS ver. 26.0.

## 3. Results

### 3.1. Distribution

We recorded 520 Chinese pangolin burrow sites along 29 transects: 70 along 13 transects in Jilongding and 7 along 2 transects in Qixingkeng, Guangdong Province; 79 along 4 transects in Xiagaoli, Jiangxi Province; and 364 along 10 transects in Putuoshan, Zhejiang Province. In addition, 372 control sites were recorded along 31 transects, including 309 along 16 transects in Jilongding and 21 along 7 transects in Qixingkeng; 4 along one transect in Xiagaoli; and 38 along 7 transects in Putuoshan. The environmental factors at each burrow and control site are provided in Supplement Table S4. Given that pangolins are at high risk for poaching, the coordinates of these sites are not disclosed.

### 3.2. Habitat Characteristics

**Figure 2:**
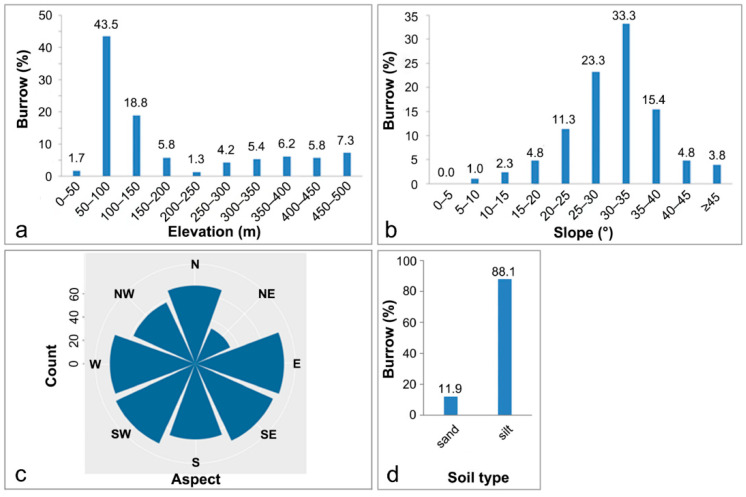
Distribution of Chinese pangolin burrows for topographic factors. (**a**): elevation, (**b**): slope, (**c**): aspect, and (**d**): soil type.

**Figure 3:**
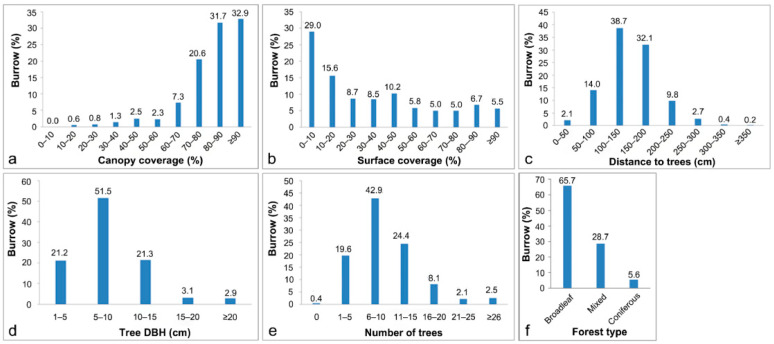
Distribution of Chinese pangolin burrows for vegetation factors. (**a**): canopy coverage, (**b**): surface coverage, (**c**): distance to trees, (**d**): tree DBH, (**e**): number of trees, and (**f**): forest type.

**Figure 4:**
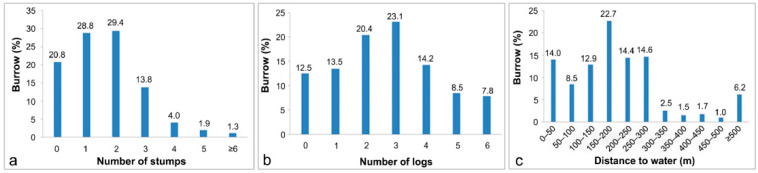
Distribution of Chinese pangolin burrows for resource factors. (**a**): number of stumps, (**b**): number of logs, and (**c**): distance to water.

**Figure 5:**
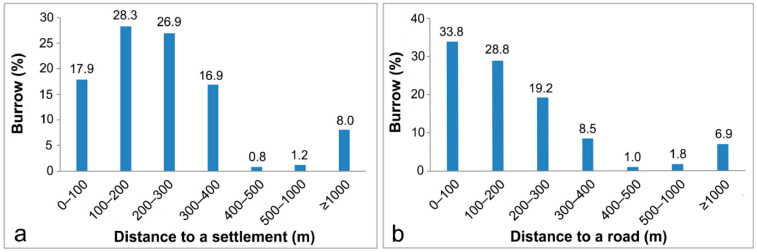
Distribution of Chinese pangolin burrows for disturbance factors. (**a**): distance to a settlement, and (**b**): distance to a road.

Burrows tended to be distributed in silty soil (88.1%) on slopes of 20–40° (83.3%) at elevations of 50–150 m (62.3%) (Figure 2a,b,d). Generally, burrows were found on all aspects, although significantly fewer were distributed on northeastern hillsides (6.3%) compared to the relatively even distribution on all other aspects (11.2–14.6%) based on the observation results (Figure 2c). Although burrows were found in all forest types, significantly more were distributed in broadleaved forests (65.8%) than in coniferous or mixed forests (Figure 3f). Within forests, the burrows were predominantly located in young and medium-aged forests with a canopy coverage exceeding 70% (Figure 3a), and were surrounded by 6–10 trees (42.9%) (Figure 3e) with a DBH of 5–10 cm (51.5%) (Figure 3d). A relatively high proportion of burrows (44.6%) was located in areas with less than 20% surface coverage, with the remaining burrows distributed fairly uniformly in environments with 20% or greater surface coverage (Figure 3b). Many burrows (86.9%) were located within 2.0 m of trees, and most were only 1.0–1.5 m from trees (Figure 3c). Many burrows (87.1%) were within 300 m of a water source (Figure 4c), and were surrounded by logs (87.5%) and stumps (79.2%) (Figure 4a,b). In terms of human disturbance, most burrows were found in forests within 400 m of a settlement (89.9%) or a road (90.4%); only a few burrows were found in areas more than 1000 m from a settlement (8.1%) or a road (6.9%) (Figure 5a,b).

### 3.3. Key Ecological Factors Affecting Habitat Selection and Distribution

Pearson correlation analyses of habitat factors showed that only distance to a settlement and distance to a road had a high correlation (r = 0.86, Supplement Figure S1). Given that pangolins are most affected by human disturbance (i.e., hunting), we chose distance to a settlement rather than to a road as the habitat factor affecting pangolin distribution for modeling. The optimal GAM included nine ecological factors: soil type, distance to a settlement, number of trees, number of logs, elevation, slope, canopy coverage, surface coverage, and tree DBH (Table 1). The cumulative residual deviation explanation rate of the nine factors was 59.9%. These results indicate that these factors drive habitat selection, while aspect, forest type, number of stumps, distance to trees, and distance to water have little influence.

**Figure 6:**
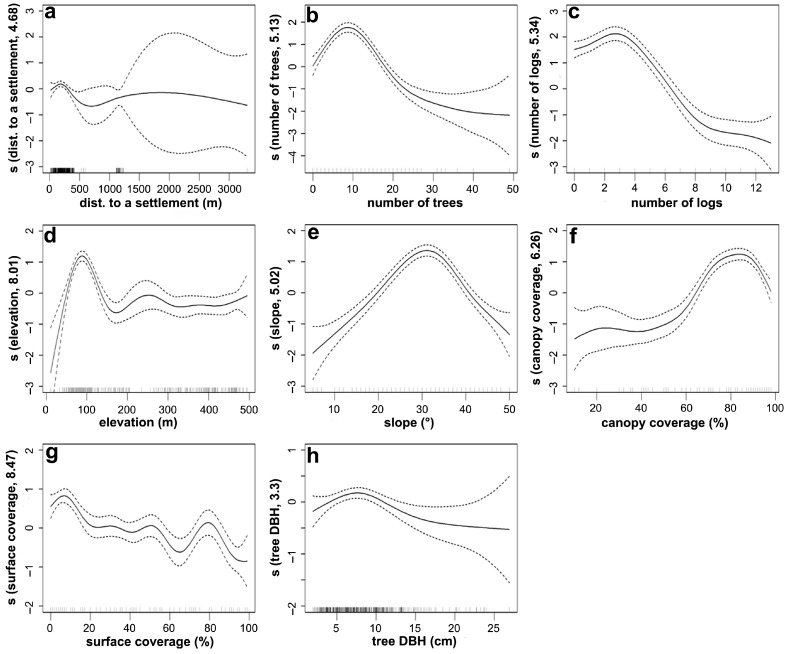
Burrow distribution of Chinese pangolins according to eight numerical key environmental factors that affect their distribution. Tick marks on the *x*-axis indicate key environmental factors, the *y*-axis represents the spline function, and dashed lines indicate 95% confidence bounds. (**a**): distance to a settlement, (**b**): number of trees, (**c**): number of logs, (**d**): elevation, (**e**): slope, (**f**): canopy coverage, (**g**): surface coverage, and (**h**): tree DBH.

The GAM fitting curves of distribution according to eight numerical key habitat factors revealed complex nonlinear relationships between the habitat factors and distribution (Figure 6). Overall, the animals tended to choose areas with a moderate elevation, slope, tree DBH, number of trees, and number of logs; there was a positive correlation with canopy coverage and surface coverage, and a weak negative correlation with distance to a settlement (Table 1).

### 3.4. Habitat Differences Among the Study Regions

**Figure 7:**
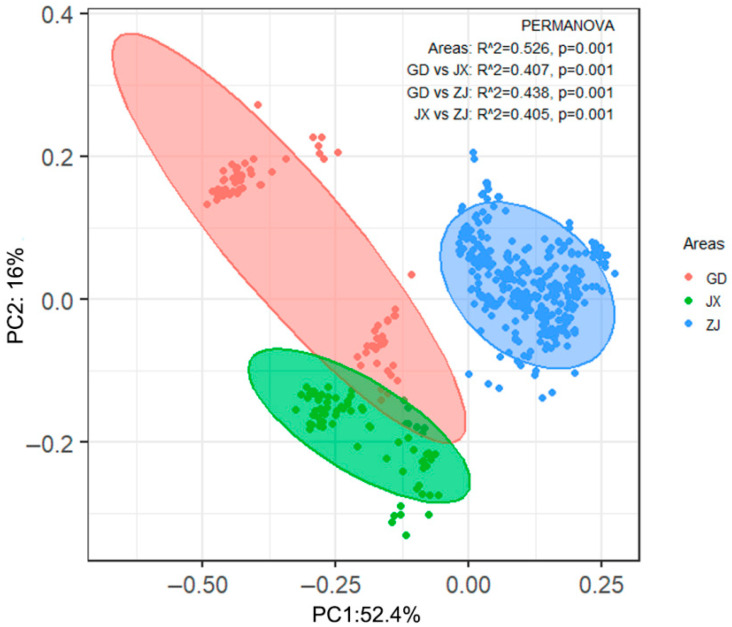
Principal coordinate analysis results of pangolin burrow habitats in Guangdong (GD; red), Jiangxi (JX; green), and Zhejiang (ZJ; blue). The shaded parts within the three circles indicate 95% confidence bounds.

**Figure 8:**
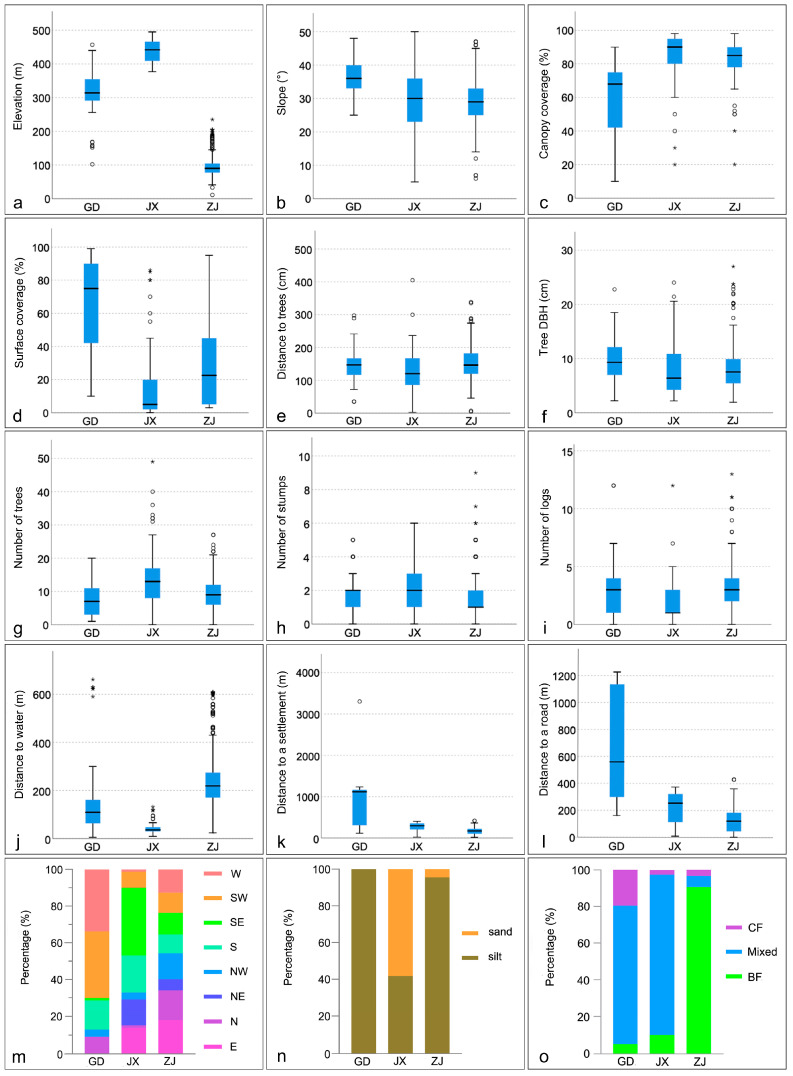
Pangolin burrow distributions for each of the environmental factors in Guangdong (GD), Jiangxi (JX), and Zhejiang (ZJ). Panels (**a**–**l**) show quartile boxplots of the various environmental factors (tick marks on the *y*-axis) shaping distribution. Panels (**m**–**o**) show percentage plots of distribution in terms of aspect (**m**), soil type (**n**), and forest type (**o**). The empty circles in the figure represent “Mild outliers”, which refer to data points located beyond 1.5 to 3 times the interquartile range. The asterisks represent extreme outliers, which refer to data points that exceed three times the interquartile range.

PCoA identified significant differences in pangolin habitat characteristics among Guangdong, Jiangxi, and Zhejiang provinces (Figure 7). We applied the Kruskal–Wallis (non-normal data, Supplement Table S2) test to analyze further the differences in 12 numerical environmental variables in the three study regions, and found significant differences in 11 of the variables (*p* < 0.05), all except the number of stumps (*p* = 0.175 > 0.05) (Table 2). The test results of independent samples showed that, except for several factors (e.g., distance to trees in Guangdong vs. Jiangxi; number of logs and distance to trees in Guangdong vs. Zhejiang; and slope and tree DBH in Jiangxi vs. Zhejiang), there were significant differences in elevation, canopy coverage, surface coverage, number of trees, and distance to water, a road, and a settlement among the three regions (Table 3). In Guangdong, burrows were found on greater slopes, with the highest surface coverage, under the lowest canopy coverage, surrounded by fewer larger-DBH trees, and farther from roads and settlements. In Jiangxi, they were located at higher elevations, under a higher canopy coverage, with a lower surface coverage, around fewer logs, closer to trees, surrounded by more small-DBH trees, and closer to water. Finally, in Zhejiang, they were located at lower elevations, farther from water, and closer to roads and settlements (Figure 8).

There were also significant differences in aspect, soil type, and forest type among the three study regions. Regarding aspect, most burrows in Guangdong were concentrated on southwestern (36.4%) and western (33.8%) slopes, with none on eastern or northeastern slopes. Although burrows were found on all aspects in Jiangxi and Zhejiang, they tended to be concentrated on southern (20.3%) and southeastern (36.7%) slopes in Jiangxi, but were relatively evenly distributed on all aspects in Zhejiang (Figure 8m). Regarding soil type, burrows were found mainly in silty soil in Guangdong (100%) and Zhejiang (95.6%), and more frequently in sandy soil (58.2%) than in silty soil (41.8%) in Jiangxi (Figure 8n). Regarding forest type, burrows were distributed mainly in mixed broadleaved forests in Guangdong and Jiangxi, and in broadleaved forests in Zhejiang (90.7%) (Figure 8o).

## 4. Discussion

Pangolins are among the most difficult wild animals to domesticate, which is thought to be related to their strict environmental requirements [36]. However, field investigations and monitoring efforts have proven difficult because of their particular morphology, habits, ecology, and sparse populations [17], resulting in a poor understanding of their habitat needs. Although there have been some studies on the habitat characteristics of Chinese pangolins, their conclusions have been inconsistent due to the inclusion of a limited number of study areas. The present study is the first to reveal the habitat characteristics of this species at a regional scale. We found significant differences among the three study regions in the habitats chosen, which could explain the lack of consistency across previous studies; our findings suggest that further investigations at larger scales are needed to clarify the habitat requirements of Chinese pangolins. Such research would enhance our understanding of its conservation needs and help identify potential occurrence sites as well as suitable reintroduction areas.

Identifying the key ecological factors that affect their distribution is an important basis for understanding their habitat requirements. Studies have generally assumed that topography, habitat type, land cover, vegetation, soil type, canopy coverage, and surface coverage are key factors affecting their distribution; some studies have also suggested that food resources, distance to a road/settlement, litter depth and distance to water determine their distribution (Table 4). In the present study, GAM analysis revealed nine key factors affecting distribution: elevation, slope, soil type, canopy coverage, surface coverage, number of trees, number of logs, tree DBH, and distance to a settlement. Tree DBH and number of logs, overlooked in prior research, emerged as key factors influencing pangolin distribution. Forests with larger tree DBH exhibit higher maturity levels. Such mature forests typically feature more complex vertical structures (such as canopy, shrub, and ground cover layers), thereby providing more diverse habitats for pangolins. While the number of logs can impact the presence of food resources such as ants and termites [29], and may also reflect food abundance.

Topographic factors are commonly considered important for Chinese pangolin habitat selection; however, studies have not formed unified conclusions regarding elevation, slope, and aspect. Different studies have reported elevation preferences as low as 100–200 m and up to 1500–1700 m; slope preferences of 5–25° versus 25–40°; and aspect preferences for all directions except north (Table 5). Our overall results imply that pangolins tend to select areas of elevation around 50–150 m on slopes of 20–40°, showing some consistency with previous studies. Perhaps in low elevation areas, due to the higher diversity and biomass of insects, pangolins have a stable food source. Moreover, its warmer nature also helps maintain the energy metabolism balance of pangolins, making it favored by pangolins. The selection of an appropriate slope gradient may be made to balance the geological risks, such as landslides or floods that are prone to occur in low elevation areas. Meanwhile, we found no clear preference for aspect overall, in contrast to previous results; however, there are significant differences in habitat characteristics among the three regions in this study. This might be due to the natural differences between different research areas, leading to significant variations in research results. This could also be the reason for the differences between the results of various studies and those of this study.

We found that pangolins were distributed mainly in forests, with the largest number of burrows located in broadleaved forests and the smallest in coniferous forests; this result significantly differs from that of a study by Wu et al. (2003) [24] in Dawuling Natural Reserve in Guangdong. When considering the results across many studies, pangolins appear to inhabit primary, secondary, and planted forests, including broadleaved, mixed, coniferous, and bamboo forests, as well as shrubland, grassland, and farmland [24,28,29,31,37]. It is understood that the tree species in the study areas of Zhejiang and Guangdong are mostly broadleaved tree species, while the main forest types in Jiangxi are subtropical evergreen broadleaved forests and coniferous forests. Therefore, we believe that the differences in forest type selection may be related to the natural differences between the research areas. Furthermore, we hypothesize that the reasons why the Chinese pangolin prefers broad-leaved forests are as follows: the broad-leaved forests have dense canopies, complex vertical space, high density of understory vegetation, and abundant shrubs and herbaceous plants, which can provide shelter and foraging space for the pangolins; the tree canopies offer shade and cooling in summer and allow light to penetrate in winter, creating a climate effect that is suitable for the survival of the warm-loving pangolins; the abundant resources in the understory increase the food diversity for the pangolins, with various types of insects. This species is widely distributed in tropical and subtropical regions of Asia, and their widespread occurrence in areas with diverse vegetation types can explain the significant differences in canopy and surface coverage within the literature. For instance, different studies have reported varying canopy coverage preferences of low coverage (0–25%), moderate coverage (26–50%), and medium to high coverage (50–75%) (Table 5), as well as surface coverage preferences of 50–75% [15,22] and 76–100% [14,24]. In the present study, burrows were distributed mainly under canopy coverages exceeding 70%, but distribution for different surface coverages was relatively balanced, albeit skewed toward a preference below 20% surface coverage. Our findings indicate that the Chinese pangolin has a preference for higher canopy coverage and lower surface coverage than suggested in previous studies, which may imply that this species can tolerate a broader range of canopy and surface coverages than previously indicated. Notably, our independent analysis of the three study regions showed that the amounts of canopy coverage and surface coverage in Jiangxi and Zhejiang were roughly consistent with the overall results, whereas the habitat in Guangdong had a lower canopy coverage (50–75%) (Figure 8c) and higher surface coverage (Figure 8d). It is worth noting that although pangolins have been found in areas with low canopy coverage and in areas with low surface coverage, this species has not been reported to occur in barren areas with both low canopy and low surface coverage. Because vegetation can affect conditions such as surface visibility, microclimate, and the abundance of ants and termites [29], we hypothesize that barren habitats would not meet the Chinese pangolin’s need for shelter, microclimate, and food resources.

Some studies have suggested that pangolins tend to avoid roads and residential areas, which might affect pangolin survival due to noise, pesticide pollution, hunting, and livestock grazing [20,24,29]. However, in contrast, we found that pangolins tended to be distributed close to roads and were not sensitive to the presence of human settlements, supporting similar findings in some areas of Nepal [21,22]. Independent analysis of the three study regions supported both of these conclusions: in Guangdong, pangolin burrows were found farther (typically more than 500 m) from human disturbances; in Zhejiang, most were within 200 m of human disturbances; and in Jiangxi (Figure 8k,l), burrows were distributed in a range that was between those of the other two sites. Around settlements, livestock grazing can negatively affect the distribution of this species [29], and free-roaming dogs can threaten their survival [29,38,39,40]. However, hunting is the major threat to pangolin survival, and most strongly impacts their distribution around settlements and roads [41]. This finding is in line with differences in pangolin hunting pressure in the three study regions. According to reports by residents of Yunnan, Guangdong, Anhui, Jiangxi, and other areas, in the 1970s and 1980s, pangolins were found around villages and often encountered on roads, but they gradually disappeared due to mass capture. In Guangdong, pangolins have been hunted and sold since the 1930s. By contrast, residents of the study area in Zhejiang reported that pangolins were not hunted historically, and that all wildlife in the area is now fully protected. In addition, studies in Fuqing City in Fujian Province [42] and Yanbian County in Sichuan Province [41] have documented the gradual shift in pangolin distribution away from residential areas due to hunting. Thus, in the absence of hunting, pangolins can survive near settlements and roads.

Although the reported preferences of Chinese pangolins for most of these ecological factors have been inconsistent, there is relatively uniform agreement on the importance of (representative) food and water resources [14,15,20,22]. For example, during our surveys, we found rich termite or ant populations in almost all burrow quadrats, supporting several other reports. The presence of logs [29] and stumps is an important factor in termite occurrence, and other studies have found that their presence is a common feature of pangolin habitat [14,43]. Moreover, most burrows have been found less than 500 m from a water source [20,21,22,23,24], in accordance with our results. Notably, our data appear to show that excessive numbers of logs and stumps were negatively correlated with pangolin distribution. However, more logs and stumps would not be detrimental to pangolin survival; rather, these two resources are limited in most environments. The general consistency of conclusions regarding food and water resources among existing studies implies that these two factors may represent the most critical determinants in pangolin habitat selection. However, due to the lack of quantitative research on ants and termites in this study, it remains unclear whether their abundances differed between burrow and control sites, and further research is warranted.

Based on our understanding of the habitat of the Chinese pangolin, there are several potential explanations for the differences among previous studies. First, the natural habitats of different study areas obviously differ, which can lead to significant differences in results. For instance, in terms of elevation, the highest elevation of some study areas was only 200 m [26], whereas the lowest elevation of some other study areas was above 1300 m [18,21]. Second, some factors may not directly influence pangolin distribution. For example, some studies have noted pangolin activity near both roads and villages [14,21,22,31,39,41], as we did in both Jiangxi and Zhejiang, whereas others have reported that pangolins maintained a distance from such areas [24,29], which was likely due to hunting by local inhabitants [24,41]. Third, research limitations inevitably play a role. For instance, field investigations typically commence in low-altitude areas, and it is difficult to venture into areas with slopes exceeding 45°. Inaccessible terrain can lead to inconsistent survey intensities within the same type of habitat among different regions. Similarly, variations in survey intensity and differences in transect design across different regions in this study may also introduce bias to the results. Therefore, the findings regarding habitat differences of pangolins among the three regions should be interpreted with caution.

Considering these aforementioned points, we propose the following. Further research on the habitat selection of this species should be carried out at local, regional, and global scales to enrich the available data. In addition, because the Chinese pangolin is a specialized predator, the richness of ants and termites within a habitat should be considered a key indicator to incorporate into distribution research. We also suggest that habitat studies should be discussed by the Pangolin Specialist Group prior to being conducted, to develop a unified research norm, including the types of habitat factors that should be included in observations and the corresponding measurement methods, so as to lay a foundation for the analysis of the habitat characteristics of this species on a global scale.

## 5. Conclusions

Chinese pangolins prefer loam slopes of 20–40°, at elevations of 50–150 m, with some trees, close to a water source (<300 m). Although this species has no strict requirement regarding canopy or surface coverage, some vegetation cover to provide shelter, an optimal microclimate, and abundant food resources is necessary for their survival. In areas where poaching is not an issue, settlements and roads do not significantly impact pangolin distribution. The habitat selection preferences of pangolins in different regions differ significantly. Global, unified research criteria should be formulated for studies on the Chinese pangolin habitat. Resource richness and the abundance of ants and termites should be taken into consideration, and the pangolin habitat at various scales should be further investigated. These factors are of great significance for a further comprehensive and accurate understanding of the habitat characteristics of this species.

## Figures and Tables

**Table 1:** Screening process of key habitat factors affecting the distribution of Chinese pangolins using a GAM based on AIC and parametric regression coefficients.

No.	Added Environmental Factors	AIC	Estimate	SE	t Value	*p* Value
1	Intercept	——	0.7836	0.0808	9.694	0.0000
2	Soil type	902.34	−0.4298	0.0282	−15.220	0.0000
3	Distance to a settlement	690.65	−0.0003	0.0000	−12.336	0.0000
4	Number of trees	558.03	0.0202	0.0023	8.703	0.0000
5	Number of logs	504.31	0.0447	0.0058	7.769	0.0000
6	Elevation	496.63	0.0004	0.0001	3.803	0.0002
7	Slope	492.32	0.0031	0.0014	2.267	0.0236
8	Canopy coverage	488.49	0.0017	0.0006	3.145	0.0017
9	Surface coverage	483.19	0.0010	0.0004	2.819	0.0049
10	Tree DBH	480.43	0.0059	0.0027	−2.170	0.0302

**Table 2:** Results of Kruskal–Wallis analysis of the differences in pangolin burrow environmental factors in Guangdong, Jiangxi, and Zhejiang.

	Elev.	Slope	Canopy	Surface	No. Logs	No. Stumps	Tree DBH	Dist. Trees	No. Trees	Dist. Water	Dist. Road	Dist. Settlement
K−W H(K)	331.639	75.739	104.693	124.359	21.459	3.489	17.282	13.904	36.757	240.537	200.124	200.549
df	2	2	2	2	2	2	2	2	2	2	2	2
Asymptotic significance	0.000	0.000	0.000	0.000	0.000	0.175	0.000	0.001	0.000	0.000	0.000	0.000

Abbreviations: Canopy, canopy coverage; df, degrees of freedom; Dist., distance to; Elev., elevation; K-W, Kruskal–Wallis; No., number of; Surface, surface coverage.

**Table 3:** Results of Kruskal–Wallis tests of the total post hoc independent samples for the environmental factors in Guangdong (GD), Jiangxi (JX), and Zhejiang (ZJ).

Environmental Factor	Adjusted Significance			Asymptotic Significance
	GD vs. JX	GD vs. ZJ	JX vs. ZJ	Total
Elevation (m)	0.004	0.000	0.000	0.000
Slope (°)	0.000	0.000	0.901	0.000
Canopy coverage (%)	0.000	0.000	0.014	0.000
Surface coverage (%)	0.000	0.000	0.000	0.000
Number of logs	0.003	1.000	0.000	0.000
Number of stumps	-	-	-	0.175
Tree DBH (cm)	0.000	0.001	0.546	0.000
Distance to trees (cm)	0.207	0.574	0.001	0.000
Number of trees	0.000	0.007	0.000	0.000
Distance to water (m)	0.000	0.000	0.000	0.000
Distance to a road (m)	0.000	0.000	0.000	0.000
Distance to a settlement (m)	0.000	0.000	0.000	0.000

**Table 4:** Key ecological factors that influence habitat selection by Chinese pangolins.

No.	Key Ecological Factors	Source
1	slope, canopy coverage, habitat type	[19]
2	aspect, slope, canopy coverage, surface coverage, soil type, distance to a settlement, habitat type, distance to water, distance to a road, ant colony	[22]
3	elevation, aspect, canopy coverage, ant colony	[30]
4	elevation, aspect, soil type	[14]
5	canopy coverage, distance to water	[29]
6	canopy coverage, soil type, distance to road, distance to a settlement	[20]
7	slope, surface coverage, distance to interference source	[24]
8	elevation, surface coverage, number of trees	[26]
9	slope, surface coverage, litter thickness, ant colony, habitat type, distance to a road	[15]
10	slope, canopy coverage, soil type, habitat type, distance to water	[23]
11	elevation, aspect, canopy coverage, soil type, vegetation form	[18]
12	canopy coverage, slope, distance to a farmland, ant colony	[21]
13	elevation, slope, canopy coverage, surface coverage, soil type, number of trees, number of logs, tree DBH, distance to a settlement	This research

**Table 5:** Habitat factors preferred by pangolins.

No.	Environmental factors	Range	Source
1	Elevation (m)	<500	[20,26]; This research
		500–1500	[15,23]
		1500–1700	[18,19,21,28,30]
2	Aspect	East	[29]
		South	[23,24]
		West	[19]
		Southeast	[22]
		Northwest	[20,22,25]
		Northeast	[14]
		Southwest	[28]
3	Slope (°)	<25	[19,20,22,23]
		25–45	[14,15,21,24]; This research
4	Canopy coverage (%)	0–25	[28]
		25–50	[14,18,22,23,30]
		50–75	[15,20,21]
5	Surface coverage (%)	50–75	[15,22,26]
		75–100	[14,24]
6	Soil type	Silt	[15]; This research
7	Habitat type	Forest	[14,19,22,23,28,29]
8	Distance to a settlement (m)	<700	[22,29]; This research
		1500–1700	[20]
9	Distance to a road (m)	<700	[21,29]; This research
		>700	[20,24]
10	Distance to water (m)	<300	[20,21,22,23,29]; This research
		300–1000	[24]
		>1000	[30]
11	Ant colony	Exist	[20,21,22]
12	Downed logs	More	[14]

## Data Availability

All the environmental factors at each burrow and control site (research data) are provided in Supplement Table S4.

## Supplementary Materials

The following supporting information can be downloaded at https://www.mdpi.com/article/10.3390/ani15142093/s1, Figure S1: Results of Pearson correlation analysis; Table S1: Transects and length in different study areas; Table S2: Environmental characteristics near Chinese pangolin burrow entrances and at control sites; Table S3: Normality test of habitat factors in three study areas; Table S4: Research data.
