# Supplementary material for: Chinese Pangolins in China Demonstrate Regional Differences in Burrow Habitat Selection
Source: Animals (Basel). 2025 Jul 16;15(14):2093. doi: 10.3390/ani15142093 (PMC12291737; doi:10.3390/ani15142093)
Supplement: Supplementary file 1 [file animals-15-02093-s001.zip › Table S1-S3.pdf]

Table S1. Transects and length in different study areas

No.	Province	Area	No. of transect	Transect length (km)
1	Guangdong	Jilongding	GD-beilingshan-01	2.2
2	Guangdong	Jilongding	GD-beilingshan-02	1.8
3	Guangdong	Jilongding	GD-beilingshan-03	1.1
4	Guangdong	Jilongding	GD-beilingshan-04	2.3
5	Guangdong	Jilongding	GD-beilingshan-05	1.3
6	Guangdong	Jilongding	GD-beilingshan-06	0.597
7	Guangdong	Jilongding	GD-beilingshan-07	1.9
8	Guangdong	Jilongding	GD-beilingshan-08	2.2
9	Guangdong	Jilongding	GD-beilingshan-09	1.2
10	Guangdong	Jilongding	GD-beilingshan-10	2.1
11	Guangdong	Jilongding	GD-beilingshan-11	1.4
12	Guangdong	Jilongding	GD-beilingshan-12	0.247
13	Guangdong	Jilongding	GD-jilongding-01	0.701
14	Guangdong	Jilongding	GD-jilongding-03	0.131
15	Guangdong	Jilongding	GD-jilongding-04	2.3
16	Guangdong	Jilongding	GD-jilongding-05	1.6
17	Guangdong	Jilongding	GD-jilongding-06	0.433
18	Guangdong	Jilongding	GD-jilongding-07	1.3
19	Guangdong	Jilongding	GD-jilongding-09	1.4
20	Guangdong	Jilongding	GD-jilongding-10	0.568
21	Guangdong	Jilongding	GD-jilongding-11	0.656
22	Guangdong	Jilongding	GD-jilongding-13	1.5
23	Guangdong	Jilongding	GD-jilongding-14	1.2
24	Guangdong	Jilongding	GD-jilongding-15	0.932
25	Guangdong	Jilongding	GD-jilongding-16	0.743
26	Guangdong	Jilongding	GD-jilongding-17	0.628
27	Guangdong	Jilongding	GD-jilongding-AA	2.7
28	Guangdong	Jilongding	GD-jilongding-AB	1.4
29	Guangdong	Jilongding	GD-tiexi-01	1.1
30	Guangdong	Qixingkeng	GD-qixingkeng-01	0.988
31	Guangdong	Qixingkeng	GD-qixingkeng-02	0.662
32	Guangdong	Qixingkeng	GD-qixingkeng-03	0.989
33	Guangdong	Qixingkeng	GD-qixingkeng-04	0.183
34	Guangdong	Qixingkeng	GD-qixingkeng-05	0.197
35	Guangdong	Qixingkeng	GD-qixingkeng-06	0.376
36	Guangdong	Qixingkeng	GD-qixingkeng-07	1.6
37	Zhejiang	Putuoshan	ZJ-putuoshan-01	1.1
38	Zhejiang	Putuoshan	ZJ-putuoshan-02	1.9
39	Zhejiang	Putuoshan	ZJ-putuoshan-03	0.504
40	Zhejiang	Putuoshan	ZJ-putuoshan-04	0.325
41	Zhejiang	Putuoshan	ZJ-putuoshan-05	0.415

42	Zhejiang	Putuoshan	ZJ-putuoshan-06	2.4
43	Zhejiang	Putuoshan	ZJ-putuoshan-07	1.3
44	Zhejiang	Putuoshan	ZJ-putuoshan-08	0.814
45	Zhejiang	Putuoshan	ZJ-putuoshan-09	2.9
46	Zhejiang	Putuoshan	ZJ-putuoshan-10	1.9
47	Zhejiang	Putuoshan	ZJ-putuoshan-11	0.327
48	Zhejiang	Putuoshan	ZJ-putuoshan-12	1.5
49	Zhejiang	Putuoshan	ZJ-putuoshan-13	0.385
50	Jiangxi	Xiagaoli	JX-xiushui-01	0.498
51	Jiangxi	Xiagaoli	JX-xiushui-02	2.4
52	Jiangxi	Xiagaoli	JX-xiushui-03	3
53	Jiangxi	Xiagaoli	JX-xiushui-04	0.709

Table S2. Environmental characteristics near Chinese pangolin burrow entrances and at control sites.

Environmental factor	Description	Methods	Unit
Elevation	Vertical distance from sea level	GPS (GPSMAP 621sc, Garmin Ltd., Olathe, KS, USA)	m
Slope	Hill angle at which burrow sites and control sites are located	Laser rangefinder (SW-50G, SNDWAY Technology (Guangdong) Co., Ltd., Dongguan, China)	degree
Aspect	Eight directions: east, northeast, north, northwest, west, southwest, south, and southeast	GPS	—
Soil type	Silt or sand (particles ≥ 2 mm in diameter accounting for ≥ 20% of the soil composition) according to particle size	Visual observation	—
Forest type	Based on the ratio of broadleaved to conifer trees within a 10 m × 10 m quadrat centered on each burrow and control site; forests were classified as coniferous forest (coniferous trees ≥ 65%), mixed forest (35% ≤ coniferous trees ≤ 65%), or broadleaved forest (broadleaved trees ≥ 65%)	Count	—
Canopy coverage	Percentage of the total projected area of the forest canopy on the ground under direct sunlight to the total area within a 10 m × 10 m quadrat centered on each burrow and control site	Visual observation	%
Surface coverage	Percentage of the total projected area of the shrub and herb canopy on the ground under direct sunlight to the total area within a 10 m × 10 m quadrat centered on each burrow and control site	Visual observation	%
Number of logs	Number of fallen logs within a 10 m × 10 m quadrat centered on each burrow and control site	Count	—
Number of stumps	Number of stumps within a 10 m × 10 m quadrat centered on each burrow and control site	Count	—
Number of trees	Average number of trees within two vertical 1 m × 5 m quadrats centered on each burrow and control site	Count	—
Distance to tree	Average distance to the tree closest to the burrow or control site within each of four 5 m × 5 m quadrats within a 10 m × 10 m quadrat centered on the burrow or control site	Laser rangefinder	cm
Tree DBH	Average diameter at breast height (DBH) of the four closest trees to burrow or control sites within each of four 5 m × 5 m quadrats within a 10 m × 10 m quadrat centered on the burrow or control site, average DBH of the four trees used to measure distance	Tree Measuring tape (Shaodong City Lijin Industry and trade Co., Ltd., Shaodong, China)	m
Distance to water	The straight-line distance measured on a map from each burrow and control site to the nearest water source	Electronic map	m
Distance to a road	The straight-line distance measured on a map from each burrow and control site to the nearest road	Electronic map	m
Distance to a settlement	The straight-line distance measured on a map from each burrow and control site to the nearest settlement/village	Electronic map	m

Table S3. Normality test of habitat factors in three study areas

Environmental factors	Area	Kolmogorov-Smirnov test (V)[a]			Shapiro-Wilk test		
		statistics	df	*P*	statistics	df	*P*
elevation	GD	.145	77	.000	.915	77	.000
	JX	.139	79	.001	.931	79	.000
	ZJ	.188	364	.000	.849	364	.000
slope	GD	.122	77	.006	.970	77	.066
	JX	.074	79	.200*	.986	79	.565
	ZJ	.095	364	.000	.972	364	.000
Canopy cover	GD	.164	77	.000	.937	77	.001
	JX	.234	79	.000	.764	79	.000
	ZJ	.132	364	.000	.899	364	.000
Herbal cover	GD	.168	77	.000	.896	77	.000
	JX	.243	79	.000	.695	79	.000
	ZJ	.185	364	.000	.875	364	.000
Fallen logs	GD	.117	77	.011	.879	77	.000
	JX	.205	79	.000	.816	79	.000
	ZJ	.182	364	.000	.888	364	.000
stumps	GD	.206	77	.000	.899	77	.000
	JX	.155	79	.000	.917	79	.000
	ZJ	.180	364	.000	.866	364	.000
Arbor DBH	GD	.085	77	.200*	.975	77	.140
	JX	.139	79	.001	.887	79	.000
	ZJ	.095	364	.000	.880	364	.000
Distance to arbor	GD	.080	77	.200*	.960	77	.017
	JX	.087	79	.200*	.939	79	.001
	ZJ	.058	364	.005	.980	364	.000
Arbor number	GD	.120	77	.008	.942	77	.002
	JX	.175	79	.000	.917	79	.000
	ZJ	.118	364	.000	.959	364	.000
Distance to water	GD	.260	77	.000	.652	77	.000
	JX	.201	79	.000	.809	79	.000
	ZJ	.171	364	.000	.847	364	.000
Distance to road	GD	.295	77	.000	.769	77	.000
	JX	.179	79	.000	.890	79	.000
	ZJ	.136	364	.000	.933	364	.000
Distance to settlement	GD	.269	77	.000	.736	77	.000
	JX	.119	79	.008	.931	79	.000
	ZJ	.071	364	.000	.980	364	.000

*: This is the lower bound of true significance;
a: Rielly's significance correction
