# Supplementary figures and images for: Chinese Pangolins in China Demonstrate Regional Differences in Burrow Habitat Selection
Source: Animals (Basel). 2025 Jul 16;15(14):2093. doi: 10.3390/ani15142093 (PMC12291737; doi:10.3390/ani15142093)

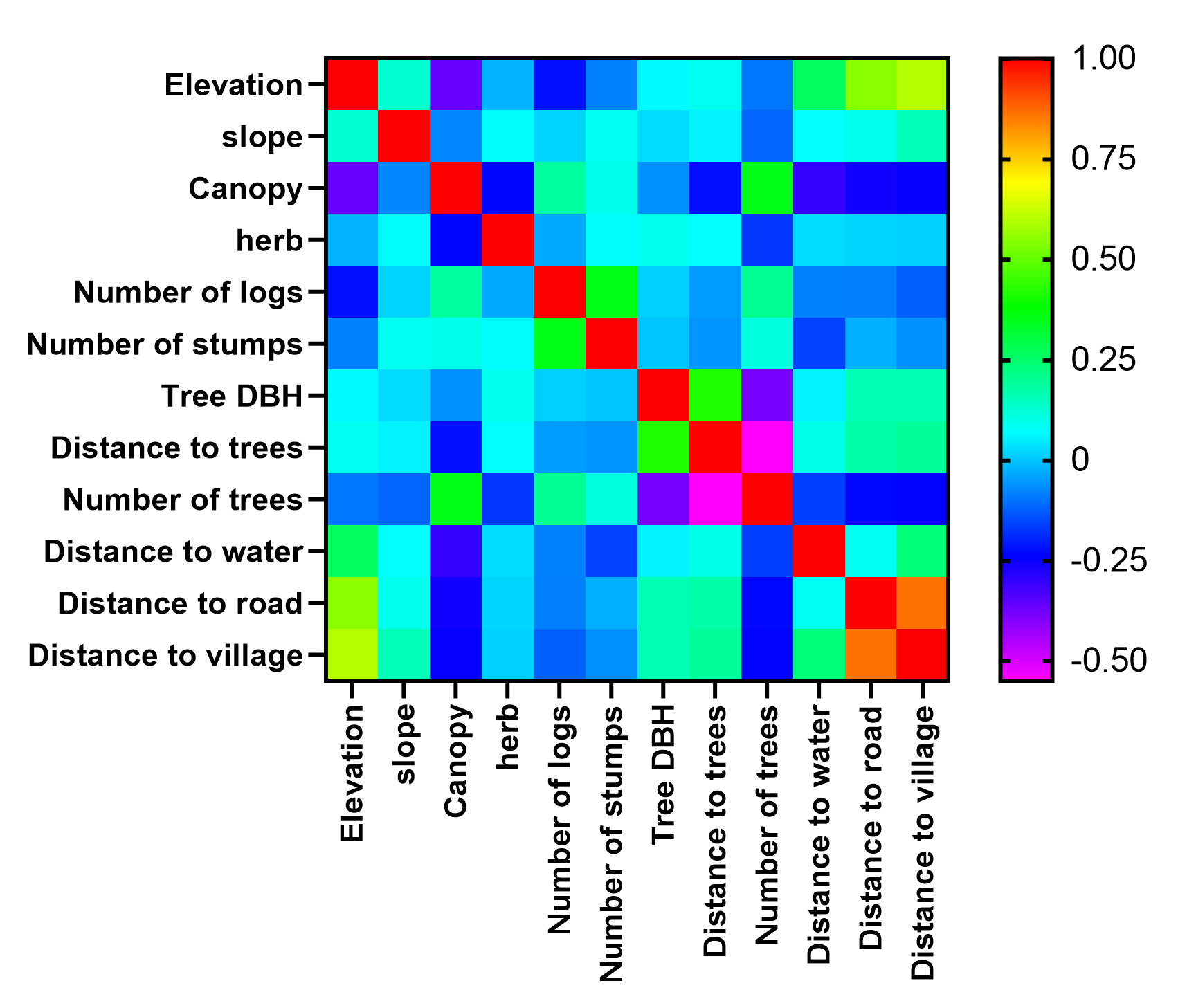

Supplement: Supplementary file 1 [file animals-15-02093-s001.zip › Figure S1. Results of Pearson correlation analysis.tif]
